# Datasets for genetic diversity assessment in a collection of wild and cultivated pomegranates (*Punica granatum* L.) by microsatellite markers

**DOI:** 10.1016/j.dib.2023.109346

**Published:** 2023-06-30

**Authors:** Angelica Giancaspro, Stefania Lucia Giove, Ilaria Marcotuli, Giuseppe Ferrara, Agata Gadaleta

**Affiliations:** Department of Soil, Plant and Food Sciences (DiSSPA), University of Bari “Aldo Moro”, Via G. Amendola 165/A, Bari 70126, Italy

**Keywords:** Pomegranate, Punica granatum, SSR, Genetic distance, Diversity, Molecular characterization

## Abstract

Data described in this article refer to molecular characterization and assessment of genetic diversity within a wide collection of pomegranate genotypes including both selections and cultivars from different geographical origin/disseminations by using microsatellite (SSR, Simple Sequence Repeats) markers. Supplied datasets refer to a set of 63 genotypes including 55 accessions (landraces) from Italy, Turkmenistan, Japan, and USA and 8 cultivars from Israel, established at the pomegranate repository of the Fruit Tree Unit of the Department of Soil, Plant and Food Science at University of Bari “Aldo Moro”, Italy. Pomegranate accessions differed for end-use purpose (edible, ornamental) and some morpho-pomological traits including juice taste, inner tegmen hardness, and skin/seed color. Molecular data were opportunely employed to build a similarity matrix to establish phylogenetic relationships (genetic similarity and distances) among pomegranate accessions and compare genetic clustering to morpho-pomological classification.

The present data article provides detailed information and methodological protocols on SSR markers, PCR amplification and banding profiling aimed to molecular characterization of pomegranate collection. This latter was conducted by amplifying a set of informative polymorphic SSR markers on the genomic DNA of each pomegranate accession, and then comparing the different molecular profiles by capillary electrophoresis. The banding patterns obtained from microsatellite markers were used to build a binary matrix containing the scores for each individual SSR fragment, which was transformed into a similarity matrix and finally used for cluster analysis and dendrogram building based on the UPGMA algorithm. This paper supplies data potentially useful for the identification of polymorphic markers suitable for varietal identification and traceability, or discrimination between tightly related pomegranate accessions with very high morphological similarity and/or geographical identity.

Data described in this paper support the published original research article titled “Exploiting DNA-based molecular tools to assess genetic diversity in pomegranate (*Punica granatum* L.) selections and cultivars” [1].

**Specifications Table**

**Table:** 

Subject	Agricultural Sciences - Agronomy and Crop Science
Specific subject area	The subject area deals with genetic diversity in a collection of fruit crop species (*Punica granatum*) including genotypes differing for some agronomical traits related to morpho-pomological features of skin, seed, inner woody tegmen, and juice.
Type of data	Tables Figures
How the data were acquired	Genetic characterization was performed on a collection of 63 pomegranate genotypes including 55 accessions (landraces) and 8 cultivars belonging to a fruit collection established at the pomegranate repository of Fruit Tree Unit of the Department of Soil, Plant and Food Science (DiSSPA) of University of Bari “Aldo Moro” (Italy) (Fig. 1). Molecular variation was assessed by using a set of 52 microsatellite (SSR, Simple Sequence Repeats) primer pairs [2]. Fruit berries had formerly been characterized for morpho-pomological and biochemical traits as described in previous works ([3,4], Fig. 2).
	The 52 SSR primer pairs used for molecular characterization [2] were derived from relevant literature in the field [5,^6^,^7^,8], and previously evaluated for their effectiveness in estimating genetic diversity within a smaller pomegranate set described in [3]. Genomic DNA was isolated from fresh young leaf samples of pomegranate plants by using the DNeasy Plant Mini Kit (Qiagen) following the manufacturer's instructions. gDNA quality was spectrophotometrically assayed at a Nanodrop device by reading the A260/A280 ratio with a value of 1.8-2.0 indicating good quality.
	Molecular data were obtained by PCR amplification of specific SSR primer pairs on 100 ng high-quality gDNA from each pomegranate accession by using M13-tailed forward primers; reactions took place in a BioRad thermal Cycler following a touch-down amplification protocol in the 60 °C-50 °C range.
	Preliminary quality and specificity check of PCR products were performed by 1.8% (w/v) agarose gel electrophoresis (Figs. 3, 4); banding patterns were visualized by running capillary electrophoresis of a 5 µL amplification reaction volume on the ABI PRISM 3500 Avant Genetic Analyzer (Applied Biosystems) automatic sequencer (Fig. 5). Electropherograms were analyzed by Gene Mapper (v. 4.7) genotyping software.
	Cluster analysis and dendrogram construction were performed by NTSYS pc v. 2.1 software [9] implementing the UPGMA (Unweighted Pair Group Method with Arithmetical averages) method, based on a genetic similarity matrix derived from the binary matrix containing the SSR scores for each pomegranate accession. In the binary matrix, each SSR fragment was treated as an independent character and scored as present (1) or absent (0) [2].
Data format	Analysed Filtered
Description of data collection	Data supplied in this article refer to a set of 63 pomegranate genotypes including 55 accessions (landraces) from Italy, Turkmenistan, Japan, and USA and 8 cultivars from Israel (Fig. 1). Fruit collection was established at the pomegranate repository of Fruit Tree Unit of the Department of Soil, Plant and Food Science (DiSSPA) at University of Bari “Aldo Moro”, Italy. Italian accessions were collected from small private orchards located in Puglia region (Southeastern Italy); the Israeli cultivars were provided by the Cairo & Doutcher farm located in Copertino (Lecce province, Puglia, Italy), and the other accessions were obtained from the USDA National Germplasm Repository in Davis (CA, USA). Fruit trees were grown at the “P. Martucci” experimental station of University of Bari located in Valenzano (Bari, Italy) equipped with environmental and soil sensors [10].
	The 63 pomegranate samples included genotypes used for both edible and ornamental purposes. Accessions differed for some morpho-pomological traits related to skin, seeds, and inner woody tegmen characteristics such as: juice taste (sweet, sour, sweet-sour), tegmen consistency (soft, hard, soft-medium), skin color (yellow-red, red, yellow, green-yellow-pink, pink-red), and seed color (white, pink, pink-red, red) (Fig. 2). Morpho-pomological and biochemical measurements were previously conducted on pomegranate fruits as reported in the work by [3], and [4].
	Microsatellites (Simple Sequence Repeats, SSR) were chosen as ideal markers for disclosing molecular variation due to their abundance, high information content, co-dominant inheritance, locus specificity, reproducibility, and easy detection, as previously reported in other works [3,^11^,^12^,13]. Genetic characterization of pomegranate accessions was carried out by using a set of 52 SSR primer pairs [2] chosen from literature [5,^6^,^7^,8] and previously assessed for their effectiveness in evaluating genetic diversity within a sub-set of pomegranate collection described in [3]. Only those primers giving specific and reliable amplicons were used for assaying genetic polymorphism within the pomegranate collection (Table 1). SSR markers were classified into “dominant” - if they amplified a single band which could be ‘present’ or ‘absent’ in the different genotypes (Fig. 3) - or “co-dominant” - if polymorphism was due to amplicons of different length (Figs. 4, 5).
Data source location	Field sample collection: • Private orchards • Puglia region • Italy • Cairo & Doutcher farm • Contrada Vigna Grande, Copertino (Lecce, LE) • Italy • Lat. 40.30238° N, long. 18.01739° E, elevation 34 m above sea level. Field data collection: • “P. Martucci” experimental station • Valenzano (Metropolitan City of Bari, BA) • Italy • Lat. 41.0438° N, long. 16.8842° E, elevation 85 m above sea level Secondary data production (analyses and filtering): • University of Bari “Aldo Moro”, Department of Soil, Plant and Food Sciences (DiSSPA) • Metropolitan City of Bari (BA) • Italy • Lat. 41.12688° N, long. 16.86596° E, elevation 5 m above sea level.
Data accessibility	Repository name: Mendeley Data Data identification number: doi:10.17632/7pwdtsn36v.2 Direct URL to data: https://data.mendeley.com/datasets/7pwdtsn36v
Related research article	A. Giancaspro, A. Mazzeo, S.L. Giove, D. Zito, I. Marcotuli, A. Gallotta, P. Colasuonno, D. Nigro, A. Blanco, N. Aradhya, A. Gadaleta and G. Ferrara, Exploiting DNA-based molecular tools to assess genetic diversity in pomegranate (*Punica granatum* L.) selections and cultivars, Fruits, 72, 5 (2017), 292-305. 10.17660/th2017/72.5.5.

## Value of the Data

• Data described in this article support original research. The paper supplies detailed methods, data, and references allowing research reproducibility.
• Datasets in this article are clearly, comprehensively, and adequately presented and are suitable to be re-used by scientific community.
• Detailed information on microsatellites markers and molecular patterns derived from their PCR amplification could be employed for genetic characterization of pomegranate collections from other geographical areas of the world. Data on genetic variability could be useful to identify SSR markers able to discriminate between synonyms and homonyms genotypes and distinguish even closely related accessions with very high morphological similarity and/or geographical identity.
• Genetic diversity assessment by microsatellite markers can supply a robust and reliable molecular tool for varietal identification. This could serve to selection of superior pomegranate genotypes to be employed directly or as donors in breeding programs for developing novel varieties endowed with improved agronomical, commercial, and nutritional properties (flavour, size, colour, antioxidant contents, disease resistance, *etc*.).
• The set of SSR markers supplied in the present data article could be employed to build binary matrices for other pomegranate collections to disclose genetic similarity/distances among genotypes and even establish any correlation between molecular and morpho-pomological features.

## 1 Objective

This dataset article was generated with the aim of collecting and comprehensively presenting all the raw data underlying the molecular characterization of a wide collection of pomegranate accessions, by using microsatellite markers. With respect to the original research to which it is referred, this data article supplies complete and detailed information related to all the tables, graphs and images in the research article, enriched with thorough technical details which are easily reusable by research community interested in pomegranate genetic diversity assessment.

## 2 Data Description

Table 1 describes the molecular data derived from PCR amplification of genomic DNA from 63 pomegranate genotypes with 37 polymorphic SSR primer pairs. For each microsatellite locus the table reports the monomorphic/polymorphic nature, the type of marker (dominant or co-dominant), the number and molecular weight of amplified SSR alleles.

**Table 1:** PCR amplification profile of 47 SSR markers on genomic DNA of 63 pomegranate genotypes including 55 accessions and 8 cultivars. For those SSR primers giving a reliable amplification product, the table reports the monomorphic/polymorphic nature in the analysed collection, the type of marker (dominant or co-dominant), and the number and molecular weight of amplified SSR alleles.

N°	SSR locus	Nature	Polymorphic type	Polymorphic alleles (N.)	Allele size (bp)
1	Pom004	monomorphic	-	0	-
2	Pom006	monomorphic	-	0	-
3	Pom010	polymorphic	co-dominant	2	248, 250
4	Pom013	polymorphic	dominant	1	368
5	Pom014	polymorphic	co-dominant	4	212, 216, 218, 221
6	Pom021	polymorphic	co-dominant	4	217, 220, 222, 251
7	Pom024	polymorphic	dominant	1	245
8	Pom039	polymorphic	dominant	1	157
9	Pom046	monomorphic	-	0	-
10	Pom055	polymorphic	co-dominant	2	263, 265
11	Pom056	monomorphic	-	0	-

12	ABRII-MP04	monomorphic	-	0	-
13	ABRII-MP07	monomorphic	-	0	-
14	ABRII-MP12	polymorphic	dominant	1	287
15	ABRII-MP26	polymorphic	co-dominant	2	180, 182
16	ABRII-MP28	monomorphic	-	0	-
17	ABRII-MP30	polymorphic	co-dominant	2	176, 192
18	POM_AAC2	polymorphic	co-dominant	4	195, 197, 201, 207
19	POM_AAC3	polymorphic	co-dominant	2	204, 206
20	POM_AAC7	monomorphic	-	0	-
21	POM_AAC12	polymorphic	dominant	1	136
22	POM_AAC13	polymorphic	dominant	1	278
23	POM_AAC14	polymorphic	dominant	1	156
24	POM_AGC5	polymorphic	co-dominant	4	119, 121, 130, 137
25	POM_AGC11	polymorphic	co-dominant	2	194, 196

26	pg1	polymorphic	co-dominant	2	235, 237
27	pg2	polymorphic	dominant	1	166
28	pg3	polymorphic	co-dominant	2	130, 136
29	pg4	monomorphic	-	0	-
30	pg5	polymorphic	dominant	1	263
31	pg6	polymorphic	co-dominant	3	206, 208, 210
32	pg7	polymorphic	dominant	1	195
33	pg9	polymorphic	dominant	1	169
34	pg11	polymorphic	dominant	1	190
35	pg12	polymorphic	dominant	1	173
36	pg13	polymorphic	co-dominant	2	169, 208
37	pg14	polymorphic	co-dominant	2	221, 244
38	pg15	polymorphic	dominant	1	204
39	pg16	polymorphic	co-dominant	3	193, 253, 268
40	pg17	polymorphic	co-dominant	4	136, 146, 148, 151
41	pg18	polymorphic	co-dominant	3	196, 202, 205
42	pg19	monomorphic	-	0	-
43	pg21	polymorphic	co-dominant	3	217, 231, 234
44	pg 22	polymorphic	co-dominant	2	245, 248
45	pg23	polymorphic	co-dominant	4	227, 237, 245, 253
46	pg24	polymorphic	co-dominant	3	91, 95, 105
47	pg 25	polymorphic	co-dominant	2	185, 206

**Fig. 1:**
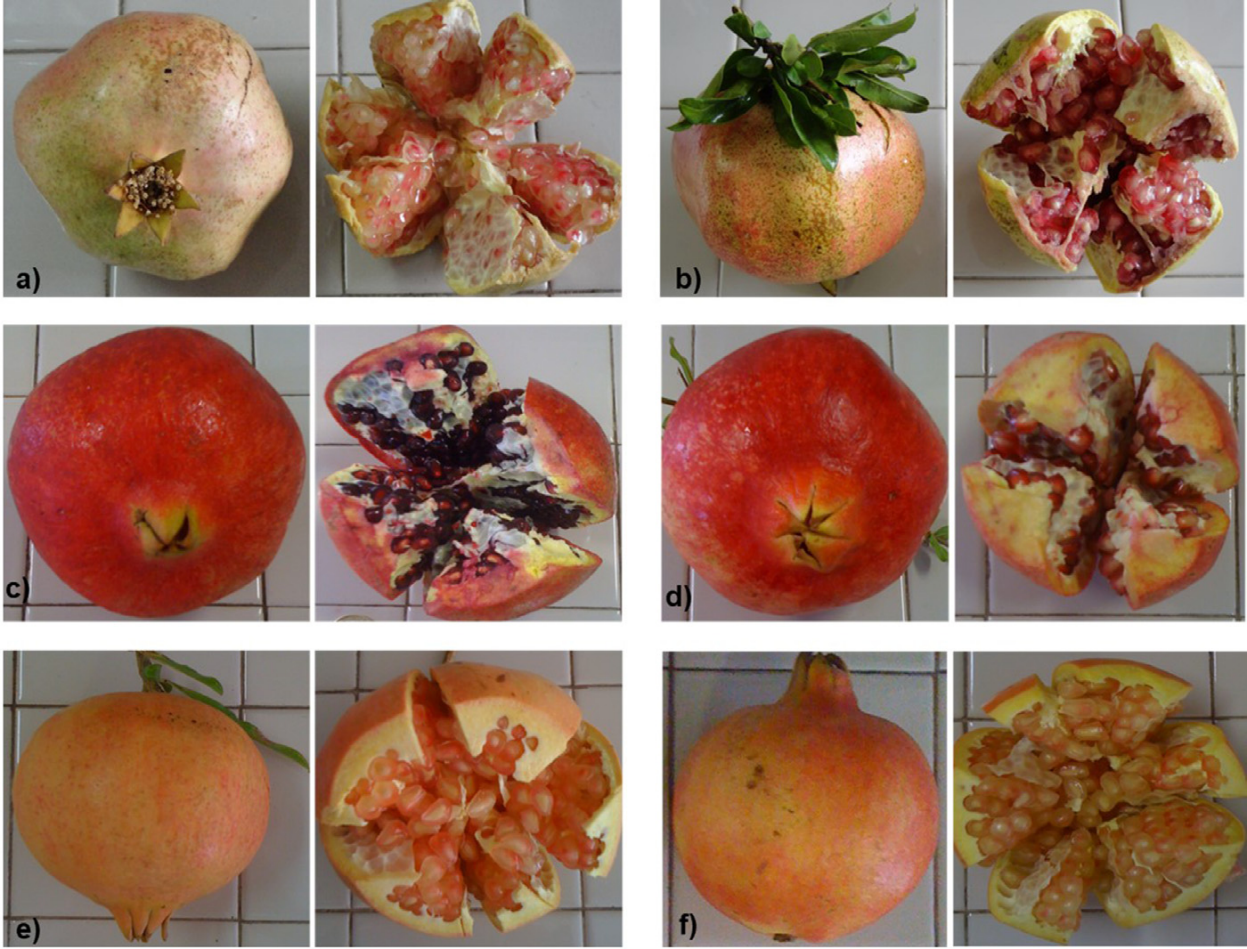
Fruit berries from six genotypes (five landraces – *lr* - and one cultivar - *cv*) of pomegranate collection at the repository of Fruit Tree Unit of the Department of Soil, Plant and Food Science (DiSSPA) of University of Bari (Italy), showing some differences for morpho-pomological traits related to skin and seeds. a) A Dente Molfetta (MG-31, *lr*); b) Locale Molfetta (MG-32, *lr*); c) Wonderful One (MG-41, *cv*); d) Ninetta (MG-51, *lr*); e) Giardino Chiuso Dolce (MG-63, *lr*); f) Maddaloni Dolce (MG-69, *lr*).

Fig. 1 supplies pictures of berries from six genotypes (5 landraces and 1 cultivar) collected from the pomegranate repository of Fruit Tree Unit of the Department of Soil, Plant and Food Science (DiSSPA) of University of Bari “Aldo Moro” (Italy) grown at the “P. Martucci” experimental station in Valenzano (Bari, Italy) [10]. Fruits show differences for some morpho-pomological traits related to skin and seeds.

Colour use is required for Fig. 1 in print.

**Fig. 2:**
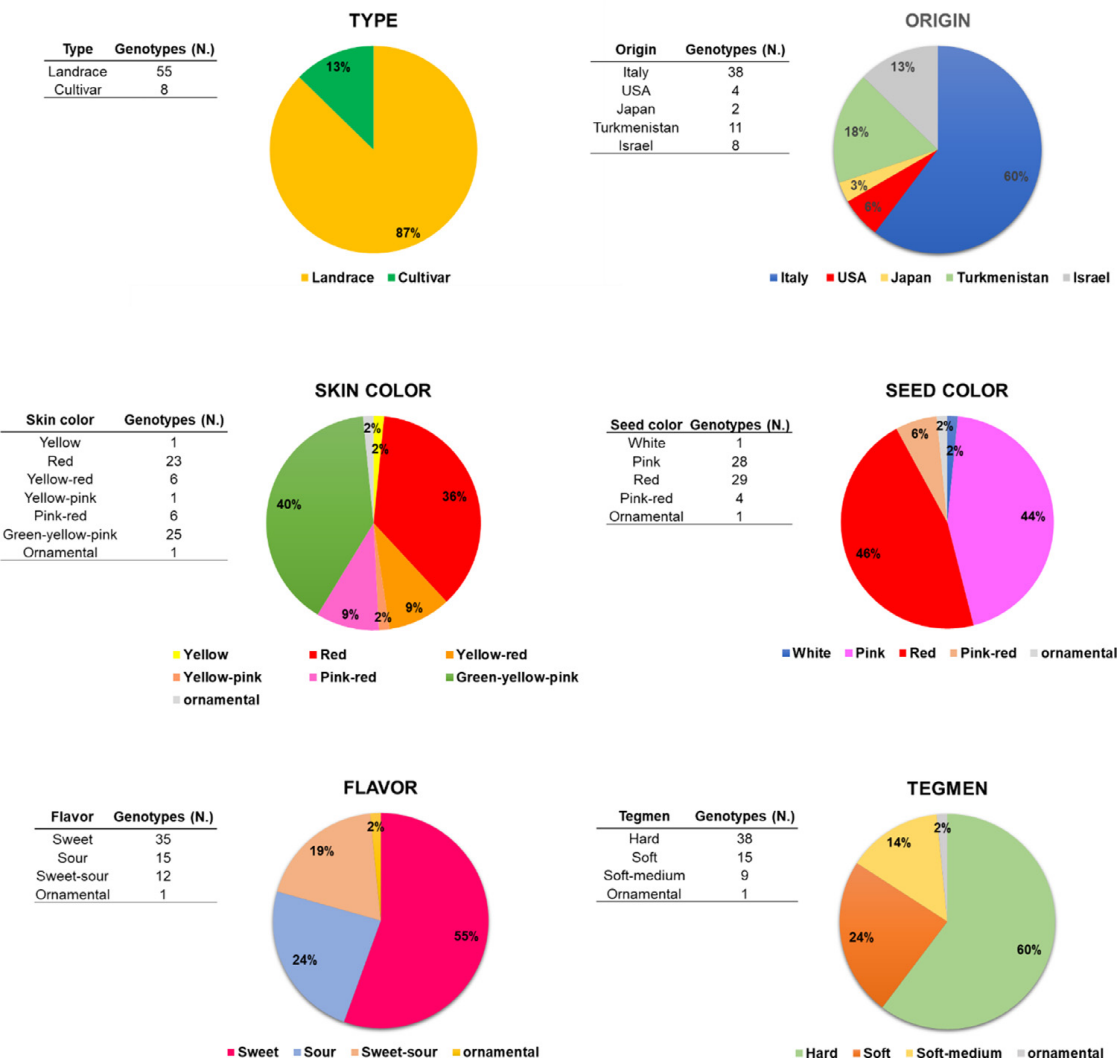
Pie charts for 63 genotypes of the pomegranate collection at the repository of the Fruit Tree Unit of the Department of Soil, Plant and Food Science (DiSSPA) of University of Bari “Aldo Moro” (Italy). Graphs depict grouping based on typology, geographical origin, and some morpho-pomological traits related to skin, seeds, and inner tegmen.

Fig. 2 reports pie charts for 63 accessions of the pomegranate collection grown at the “P. Martucci” experimental station of University of Bari “Aldo Moro” located in Valenzano (Bari, Italy) [1,10]. Grouping is based on geographical origin (or centers of diffusion) and some morpho-pomological traits relative to skin, seeds, and inner woody tegmen (juice flavor, skin and seed color, tegmen hardness).

**Fig. 3:**
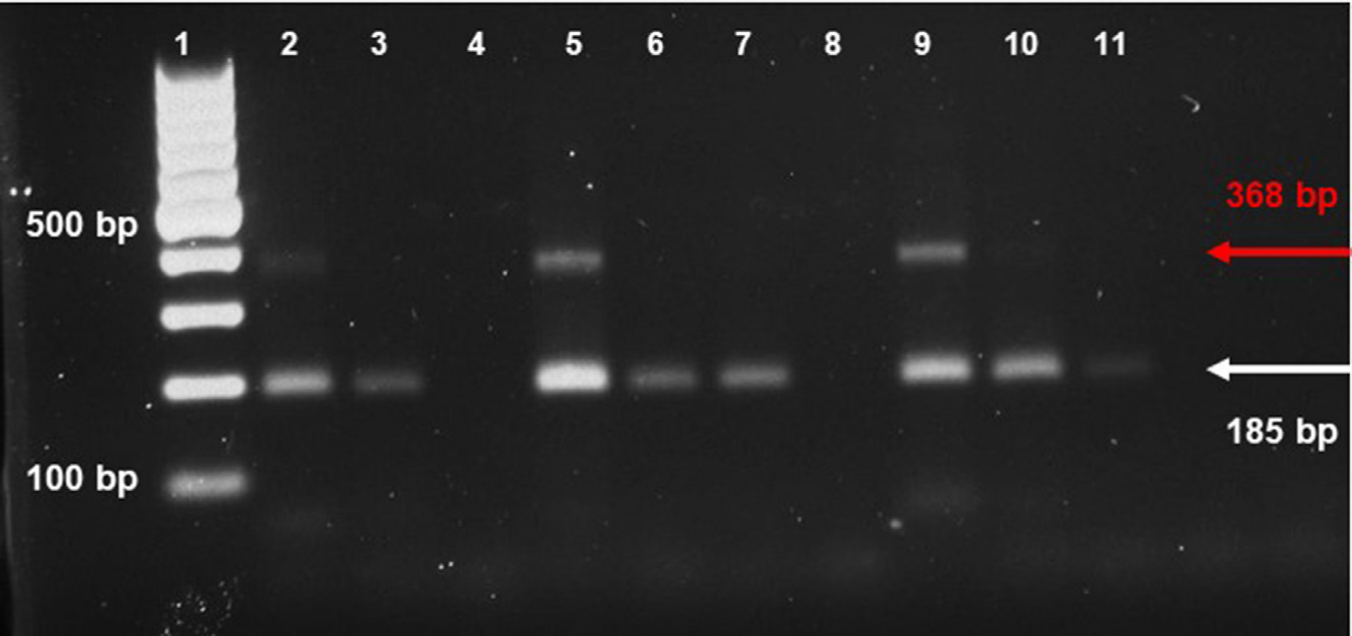
Electrophoretic pattern on 1.8% (w/v) agarose gel of PCR-amplified fragments from SSR marker “pom013” on a sub-set of pomegranate genotypes. Lane 1: 100 bp DNA ladder; lanes 4 and 8: empty lanes with no-amplification product; lanes 2, 3, 5-7, 9-11: lower-molecular-weight band representing a monomorphic SSR allele; higher-molecular-weight band representing a polymorphic SSR allele with a dominant pattern (presence/absence).

Fig. 3 depicts the electrophoretic pattern on 1.8% (w/v) agarose gel of PCR amplification products of polymorphic microsatellite marker “pom013” on a sub-set of pomegranate genotypes. The marker is composed by a lower-molecular-weight band representing a monomorphic SSR allele, and a higher-molecular-weight band representing a polymorphic allele with a dominant pattern (presence/absence).

**Fig. 4:**
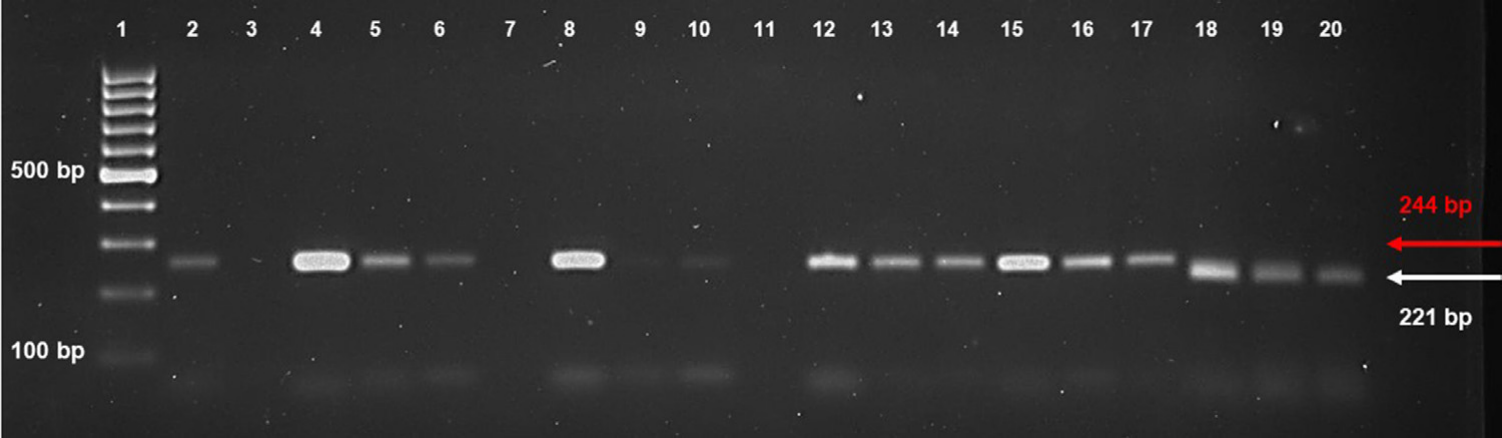
Electrophoretic pattern on 1.8% (w/v) agarose gel of PCR-amplified fragments from SSR marker “pg14” on a sub-set of pomegranate collection. Lane 1: 100 bp DNA ladder; lanes 3, 7, 11: empty lanes with no-amplification product; lanes 2, 4-6, 8-10, 12-20: SSR marker showing a polymorphic co-dominant pattern (two different allele lengths).

Fig. 4 reports the electrophoretic pattern on 1.8% (w/v) agarose gel of PCR amplification products of polymorphic microsatellite marker “pg14” on a sub-set of pomegranate accessions. The SSR marker shows a co-dominant pattern profiled by two alleles with different molecular weight.

**Fig. 5:**
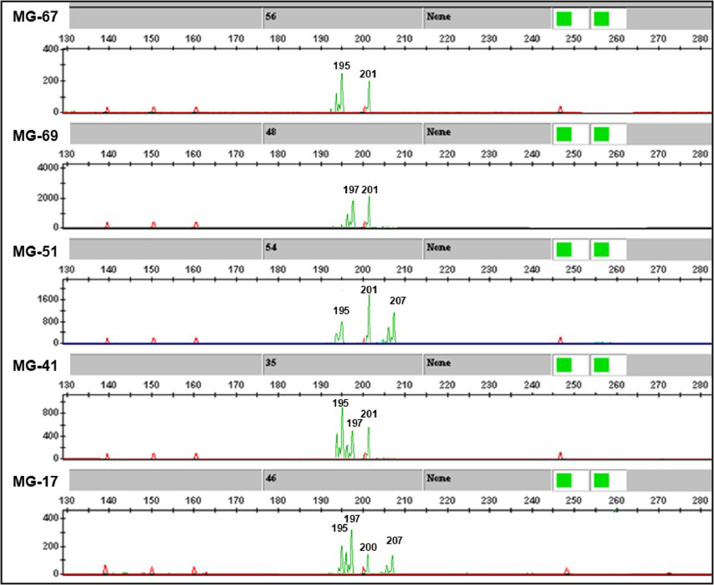
Electrophoretic patterns obtained by capillary electrophoresis of PCR-amplified alleles of “pom-AAC2” SSR co-dominant marker in five pomegranate genotypes (four landraces, *lr*, and one cultivar, *cv*). Green peaks derive from M-13 tailed SSR primers labelled with HEX fluorophore. Red peaks refer to an internal size standard labelled with ROX fluorophore. MG-67: Fiore Mola Acido, *lr*; MG-69: Maddaloni Dolce, *lr*; MG-51: Ninetta, *lr*; MG-41: Wonderful One, *cv*; MG-17: Acido Torre Canne, *lr*.

Fig. 5 shows the electrophoretic patterns obtained by capillary electrophoresis of PCR-amplified alleles from “pom-AAC2” microsatellite marker in five pomegranate genotypes (four accessions and one cultivar). The electropherogram depicts an informative polymorphic SSR marker characterized by a co-dominant nature.

Supplementary Table 1, publicily available at the Mendeley Data repository https://data.mendeley.com/drafts/7pwdtsn36v (doi:10.17632/7pwdtsn36v.2) lists 52 microsatellite primer pairs used to assay genetic diversity within a pomegranate collection of 63 genotypes including 55 accessions (landraces) and 8 cultivars [1] from the Fruit Tree Unit of the Department of Soil, Plant and Food Science (DiSSPA) of University of Bari (Italy). The table reports the name, sequences of forward and reverse primers, repeat motif, reliability, and bibliographic reference for each SSR marker. The table also contains the scores relative to 77 informative SSR markers (derived from PCR amplification of 37 polymorphic SSR primer pairs) employed for cluster analysis and estimation of genetic distances among the 63 pomegranate genotypes. For all pomegranate accessions, each amplified SSR allele is annotated with the score “1” (present), “0” (absent) or “9” (missing data). SSR scores were converted into a binary matrix implemented into NTSYSpc v.2.1 software for dendrogram building.

## 3 Experimental Design, Materials and Methods

Datasets supplied in this article refer to the assessment of genetic diversity and phylogenetic relationships within a comprehensive collection of 63 pomegranate genotypes including 55 accessions (landraces) from Italy, Turkmenistan, Japan, and USA and 8 cultivars from Israel [1]. Italian accessions were collected from private orchards located in Puglia region (Southeastern Italy), whereas the Israeli cultivars were provided by the Cairo & Doutcher farm located in Copertino (Lecce province, Puglia, Italy); the remaining accessions were obtained from the USDA National Germplasm Repository in Davis (CA, USA). Fruit collection was established at the pomegranate repository of the Fruit Tree Unit of the Department of Soil, Plant and Food Science (DiSSPA) of University of Bari “Aldo Moro”, Italy. Fruit trees were grown in Valenzano (Bari, Italy) at the “P. Martucci” experimental station equipped with environmental and soil sensors [10]. The 63 pomegranate samples included genotypes used for both edible and ornamental purposes and differed for some morpho-pomological traits related to skin, seeds and inner tegmen such as: juice taste (sweet, sour, sweet-sour), tegmen consistency (soft, hard, soft-medium), skin color (yellow-red, red, yellow, green-yellow-pink, pink-red), and whole seed color (white, pink, pink-red, red) (Fig. 1, 2). Morpho-pomological measurements were previously conducted on pomegranate fruits as reported in the works by [3,4].

Genomic DNA was isolated from 100 mg of fresh young leaf samples of pomegranate plants by using the DNeasy Plant Mini Kit (Qiagen) following the manufacturer's instructions. DNA quality was spectrophotometrically checked at a Nanodrop device by reading the A260/A280 ratio with a value of 1.8-2.0 indicating good quality. Genomic DNA of all samples was adjusted to a 25 ng/µL final concentration to be used in following PCR reactions. Amplifications of microsatellite markers were performed on 100 ng high-quality gDNA from each pomegranate accession, by using a set of 52 SSR primer pairs derived from relevant literature in the field [2,^5^,^6^,^7^,8]. A PCR reaction volume of 12.5 µL containing 25 ng of gDNA template, 0.032 µM of M13-tailed forward primer, 0.16 µM of reverse primer and 0.8 µM of Fam- or Hex-labelled M13 tail, 0.2 mM of each dNTP, 2 mM MgCl2, 1X PCR Buffer (10 mM Tris-HCl, pH 8.3; 10 mM KCl), and 0.5 unit of Taq DNA polymerase (Euroclone, EuroTaq) was set to amplify SSR markers. Reactions were performed in a BioRad thermal cycler according to the following amplification protocol: 5 min at 95 °C followed by 20 touchdown cycles of: 45 s at 95 °C, 1 min at 60 °C (0.5 °C lower per cycle) and 1 min at 72 °C, and 25 cycles of: 45 s at 95 °C, 1 min at 50 °C and 1 min at 72 °C, with a final extension step of 7 min at 72 °C.

Amplification products were preliminarily checked for size and quality by a standard electrophoresis on 1.8% (w/v) agarose gel (Figs. 3, 4), thus only the primer pairs giving clear, specific, and reliable amplicons were used for assaying genetic polymorphism within the whole pomegranate collection (Table 1). SSR fragments contained in a 5 µL reaction volume for each accession were separated by capillary electrophoresis performed on an ABI PRISM 3500 Avant Genetic Analyzer (Applied Biosystems) automatic sequencer, and the corresponding genetic profiles drawn by analyzing electropherograms by Gene Mapper v.4.7 genotyping software (Fig. 5).

Only primer combinations giving specific polymorphic amplification products were employed for genetic characterization of pomegranate collection (Table 1). Among these latter, SSR markers were classified into “dominant” - if they amplified a single band which was present or absent in the different genotypes - or “co-dominant” - if their polymorphism was due to a different amplicon length (Table 1, Figs. 3, 4, 5). In all cases, null alleles were confirmed by running PCR on three technical replicates of the same sample. For each amplified SSR locus, a direct scoring of the allele size (molecular weight) was firstly performed, then results were converted in a binary matrix in which each SSR fragment was treated as an independent character and scored as present (1), absent (0) or missing (9) [2]. A genetic similarity matrix was computed from the binary matrix by using the Jaccard's coefficient in pairwise comparisons, then cluster analysis and dendrogram construction were performed by NTSYSpc v. 2.1 software [9] implementing the UPGMA (Unweighted Pair Group Method with Arithmetical averages) method.

## Ethics Statements

The work meets the ethical requirements for publication in Data in Brief. The work does not involve studies with animals and humans.

## CRediT authorship contribution statement

**Angelica Giancaspro:** Methodology, Investigation, Formal analysis, Visualization, Data curation, Writing – original draft. **Stefania Lucia Giove:** Methodology, Investigation, Formal analysis. **Ilaria Marcotuli:** Investigation. **Giuseppe Ferrara:** Conceptualization, Resources, Project administration, Funding acquisition, Supervision, Writing – review & editing. **Agata Gadaleta:** Conceptualization, Supervision, Writing – review & editing.

## Declaration of Competing Interest

The authors declare that they have no known competing financial interests or personal relationships that could have appeared to influence the work reported in this paper.

## Data Availability

Microsatellites markers used to assay genetic diversity in a pomegranate collection of 63 genotypes including 55 landraces and 8 cultivars (Original data) (Mendeley Data).

## Appendix Supplementary materials

Image, application 1
